# Scholarly communications program: force for change

**DOI:** 10.1186/1742-5581-3-6

**Published:** 2006-06-21

**Authors:** Barbara M Koehler, Nancy K Roderer

**Affiliations:** 1 William H. Welch Medical Library, Johns Hopkins University, 1900 East Monument Street, Baltimore, MD 21205, USA

## Abstract

The changing landscape of scholarly publication and increasing journal costs have resulted in a need for proactive behavior in libraries. At Johns Hopkins University in Baltimore, Maryland, a group of librarians joined forces to bring these issues to the attention of faculty and to begin a dialog leading to change. This commentary describes a comprehensive program undertaken to raise faculty awareness of scholarly communications issues. In addition to raising faculty interest in the issues at hand, the endeavor also highlights an area where library liaisons can increase their communication with the units they serve.

## Background

There has long been consensus that there is a crisis in scholarly communications. The reasons are evident: rising costs for scholarly materials, particularly journals; stable or declining university budgets; declining numbers of society publishers providing reasonable pricing; mergers within the commercial publishing industry resulting in less competition and increased prices; and a shifting emphasis from communicating scientific information to generating profits for publishing company stockholders. Decades of double-digit increases in journal prices coupled with decreasing support for library budgets have presented powerful dilemmas for librarians and universities. At the same time that prices were rising and budgets were falling short, a monumental shift in publishing was moving materials from print to electronic. Librarians hoped to see reductions in journal costs as a result of the shift, but in fact saw continuing increases. There was lively interest in exploring alternatives to re-invent the world of scholarly publishing.

As early as 1997, there was formal agreement within the University of California system that the current model of scholarly communication was unsustainable. [1] The University Library Committee of the Faculty Senate of the University of Wisconsin (UW) addressed the topic in its Annual Report for 1998–99, stating that one of the issues of overriding importance to the future of the UW-Madison libraries was "the future of academic publishing, intellectual property rights, and alternative publication outlets for scholarship." [2] The Association for Research Libraries (ARL) addressed scholarly communications issues with the launch of SPARC, the Scholarly Publishing and Academic Resources Coalition, in June 1998 with the goal of providing a constructive response to a dysfunctional marketplace. [3] In January 2002, the board of the Association of College & Research Libraries (ACRL) authorized a Scholarly Communications Initiative which would enable ACRL to play a prominent role in bringing about change in the scholarly communications system. [4] During this same period of time, many academic libraries at universities as diverse as Cornell, Georgetown, University of Florida, University of Utah and the University of California produced exhaustive websites on the subject. At least ten universities across the United States have publicly affirmed their support for change in campus-wide resolutions. [5]

The issues began to coalesce into a coherent narrative at Johns Hopkins University with the arrival of James G. Neal as Sheridan Director of the Milton S. Eisenhower Library in the fall of 1995. Founded in 1876 in Baltimore, Maryland, the Johns Hopkins University was the first research university in the United States. Its aim is not only to advance students' knowledge, but also to advance human knowledge generally, through discovery and scholarship. The university's emphasis on both learning and research – and on how each complements the other – revolutionized U.S. higher education. Today, Johns Hopkins has campuses throughout the world – China, Italy and Singapore, among many others. It remains a world leader in teaching, patient care and discovery. The Milton S. Eisenhower Library on the Homewood campus houses the major university collections of over 2 million volumes and over 9000 subscriptions to current journals. The Welch Medical Library on the East Baltimore medical campus serves the schools of medicine, public health, and nursing as well as the Johns Hopkins Hospital. Its services and collections are largely electronic with subscriptions to nearly 6000 e-journals, e-books and databases.

Jim Neal had been actively involved in scholarly publishing and copyright issues for much of his career. During his years at Hopkins, he worked to educate his own faculty about the issues, regularly briefing his management team and using the Faculty Advisory Committee for the Libraries to expand on issues he felt were critical. Neal's early and intense involvement in scholarly communications issues led to a heightened awareness among Hopkins librarians. Library staff began to read widely on the subject, discovering what their own professional associations were doing as well as noticing brochures and websites created by concerned groups such as Creative Commons. Librarians were good at aggregating a lot of data, but how could they share it with the people who needed to know, Hopkins faculty?

## Discussion

The first conversations at the Welch Library took place in 2001. Led by Welch Library director, Nancy Roderer, a forum was held under the auspices of the Hopkins Libraries to raise awareness of scholarly communications issues. A keynote address by David E. Schulenberger, provost of the University of Kansas, was followed by addresses from Jim Neal, by then President of Information Services and University Librarian at Columbia University, and three Hopkins faculty. [6] Ms. Roderer subsequently convened a monthly meeting of a Scholarly Communications Group (SCG) composed of interested librarians from all of the Hopkins libraries. The SCG operates under the aegis of the University Library Committee chaired by the Dean of University Libraries and Director of the Sheridan Libraries, Winston Tabb. Tabb himself has been an ardent supporter of scholarly communications initiatives, speaking publicly on the issues on a regular basis. The SCG's goal was to capitalize on the emerging interest fueled by the symposium to educate faculty about the issues and to encourage appropriate actions. Everyone agreed that the approach must be practical – actions that interested parties could implement easily.

### First steps

Many universities already had scholarly communications websites devoted to issues and actions, among them University of California, Cornell, Massachusetts Institute of Technology, University of Washington, and North Carolina State University. With the help of the Welch Library's Advanced Technologies and Information Systems group, the Hopkins SCG completed the design of its website . The primary function of the site is to promote awareness of issues, initiatives, and practices while offering an interactive space for users' responses. Issues are clearly spelled out on the site followed by a call to action. One of the most ambitious sections of the site, the Author's Tool, provides a list of electronic journals linked to a wealth of information about each such as the publisher, the impact factor, publishing instructions, Hopkins' usage statistics, etc. An interactive section allows authors who have published in particular journals to comment on their own experiences, good or bad, and alert their colleagues to certain publishers' practices. Users can find a wealth of information on the site under headings such as Organizations and Institutions, New Publishing Models, and Readings. A News and Events column summarizes current articles on relevant subjects. The site is visited by users both within and outside of Hopkins.

Building on the usefulness of the website, the group turned its attention to letting people know of its existence through a brochure about it. Both the Welch Library on the medical campus and the Eisenhower Library on the main campus have strong outreach or liaison programs. These programs respond to the distributed and complex environments of campuses where library users employ technology in their homes and offices and do not visit the library buildings. Liaisons are assigned to various departments and regularly ask them how they are using information and what they need from libraries to enhance their search for and use of information. Through outreach endeavors – classes, orientations, desk encounters – the scholarly communications brochures were made available to faculty, staff and students. When liaisons meet with their assigned departments, they also mention the scholarly communications initiatives, handing out brochures and answering questions. These meetings provide another opportunity to market concerns about the changing world of publishing.

Staff believed they could engage authors in a useful dialog about scholarly communications if they could identify the right people. In 2004, driven by an upcoming negotiation with Elsevier, one of the largest commercial publishers of scientific, technical and medical material, the SCG built a database of Hopkins Elsevier authors which was used to contact some of these key players – prolific authors, influential faculty and important journal editors – explaining the issues, and asking for their support in negotiating with Elsevier for the option of choosing the titles we wanted, not a "big deal" package of all the titles available in certain subject areas. Faculty agreed to support this.

### Engaging the faculty

In an effort to reach busy Hopkins faculty preoccupied with their own research, the SCG took the advice of the Welch Library Advisory Committee who urged them to take the case directly to individual departments. The Advisory Committee suggested a slide show and talk that would prompt interest and discussion in a congenial group setting. Other universities had held similar meetings with small groups of faculty, among them the University of California (UC) who had held scholarly communications faculty seminars in fall 2003. Originating in the Academic Council, the seminars were developed to explore issues between UC faculty, university librarians, and systemwide committees involved with scholarly communications. [7] On the main Hopkins campus, director Winston Tabb delivered the talks; on the medical campus, SCG member Barbara Koehler developed and presented a slide show and talk. The appropriate liaison accompanied the speaker to each faculty meeting, answered any questions that arose and handled follow-up questions. Thus, the faculty heard and saw what was going on in the world of scholarly publishing, and the session would have the additional benefit of raising the profile of the assigned liaison. Nine slides led the audience from issues of costs and markets, crises and threats, to an action plan centered on what the library is doing and what the faculty can do to address the critical issues. Most departments readily agreed to a 10 to 20 minute presentation and often peppered staff with questions about scholarly issues as well as about other library concerns. By February 2006, more than 30 talks had been presented, and the plan is to continue these presentations until all departments have heard the message.

What has piqued the interest of listeners? They are, of course, interested in their careers and in publishing in prestigious journals, so they often comment on the issue of selecting open access or author-friendly journals whose titles might be less prestigious. They frequently ask about retaining their own copyrights – exactly how to do it or what language to use. They are appalled to find out that often they have to get permission, or worse, pay, to use their own work. They are surprised to find that the library often has to pay to put their work on electronic reserve for a class. If they are members of a society, they may express dismay at the open access movement because of their concern for the economic underpinnings of society publishing. Often people are surprised at the subscription prices for certain journals. Some departments are energized by the talks and ask specifically what they can do to help. People ask about the impact of new open access journals: are they being read and cited? Some faculty openly express fear of any change in the current system of journal publishing, concerned that change might lead to the destruction of a system that, in their view, has worked well for many years.

The departmental talks made it easy for us to communicate information about the National Institutes of Health (NIH) open access effort and make our support known to faculty. In May 2005, the NIH requested that federally-funded research articles be made available to the public via deposit in PubMed Central within 12 months of publication. The initiative was of interest though many faculty feared its impact or were confused about making submissions. We talked about the issues with them and also included information about the NIH plan on our website. Eventually, we met with the research deans for three Hopkins schools, presenting them with text we had drafted on the NIH proposal and asking them to include it on their own research sites, which they agreed to do.

Have the talks made a difference? Increasing calls to the library relating to issues introduced in our talks tell us that we have succeeded in raising awareness. Additionally, members of the SCG have been invited to participate in focus groups for several publishers – the National Academy of Sciences, the American Society of Hematology, the Federation of American Societies for Experimental Biology, and the Society for Plant Biologists – an indication that issues raised within Hopkins and other universities are beginning to have a wider impact in the publishing world. A recent survey from the Centre for Information Behaviour and the Evaluation of Research reports that there has been a rise in the number of authors who know something about open access (OA) and a decrease in the number who know nothing at all. Over the same year, the number of authors publishing in OA journals has grown. [8] These figures seem to indicate that overall awareness is growing, and we believe we are contributing to that.

With scholarly communications talks underway, members of the SCG decided to capitalize on the interest generated to reinforce the discussion in another way. A grant from the National Network of Libraries of Medicine/Southeastern/Atlantic Region funded a forum devoted to scholarly communications issues, jointly organized by the Johns Hopkins University Libraries and the University of Maryland, Baltimore, Health and Human Services Library. Entitled, "Ownership and Access in Scholarly Publishing," the forum featured presentations by Hopkins faculty as well as representatives from the National Institutes of Health, the National Academy of Sciences, and Science Commons. There were multiple venues for the talks and the forum was webcast. [9]

## Conclusion

Scholarly communication is approaching a crossroads. If the current system is unsustainable, as many believe, and if technology has changed the landscape of publishing, then a time for serious decision-making is at hand. There is a role for the libraries and librarians in this enterprise – continuing to support authors and to disseminate scholarly communications information until there is an economically sustainable system that provides the widest possible access to scholarship. The goal of the Hopkins' scholarly communications initiative has never been to undermine the world of scholarly publishing. It is not necessary to make everything free for libraries or to put publishers out of business. Indeed, our goals are to ensure that Hopkins authors know what their rights are, that they manage their own work in a way that benefits science as well as their own needs, that they understand the business plans and philosophy of the journals they work for, and that they take control of their own publishing destinies.

## Competing interests

"In 2005, I co-authored a *JMLA *article with Nancy Roderer, Director of the Welch Medical Library, who is mentioned in the present article. I do not believe that this, in light of my reviewing the present submission, constitutes a conflict of interest. I feel compelled nonetheless to disclose personal familiarity with some activities of Welch librarians."

Jerry Perry, Reviewer

## References

[B1] Library Planning and Action Initiative. http://www.slp.ucop.edu/lpai_new/finalrpt/report.html#E.

[B2] Annual Report of the 1998–99 University Library Committee. http://www.library.wisc.edu/libraries/News/ULC/reports/98_99.pdf.

[B3] About SPARC. http://www.arl.org/sparc/about/index.html.

[B4] English, Ray ACRL Scholarly Communications Initiative: a Progress Report. http://www.ala.org/ala/acrl/acrlpubs/crlnews/backissues2004/september04/scholarlycomminitiative.htm.

[B5] Faculty Speak Out About Scholarly Communication Issues. http://www.arl.org/osc/faculty/index.html.

[B6] Science Publishing Alternatives: A Forum. http://www.welch.jhu.edu/publish/pls.html.

[B7] Reshaping Scholarly Communication. http://osc.universityofcalifornia.edu/responses/faculty_forums_bg.html.

[B8] Rowlands I, Nicholas D New Journal Publishing Models: an International Survey of Senior Researchers. http://www.slais.ucl.ac.uk/papers/dni-20050925.pdf.

[B9] Ownership & Access in Scholarly Publishing. http://www.openaccess.umaryland.edu/webcast.html.

